# Multimeric Association of Purified Novel Bowman-Birk Inhibitor From the Medicinal Forage Legume *Mucuna pruriens* (L.) DC.

**DOI:** 10.3389/fpls.2021.772046

**Published:** 2021-11-25

**Authors:** Jafar K. Lone, Mandapanda A. Lekha, Rajiv P. Bharadwaj, Fasil Ali, M. Arumugam Pillai, Shabir H. Wani, Jeshima Khan Yasin, K. S. Chandrashekharaiah

**Affiliations:** ^1^Department of Studies and Research in Biochemistry, Mangalore University, Konaje, India; ^2^Department of Plant Breeding and Genetics, Agricultural College and Research Institute, Tamil Nadu Agricultural University, Tuticorin, India; ^3^Mountain Research Centre For Field Crops, Sher-e-Kashmir University of Agricultural Sciences and Technology of Kashmir, Srinagar, India; ^4^Division of Genomic Resources, Indian Council of Agricultural Research (ICAR)-National Bureau of Plant Genetic Resources, New Delhi, India

**Keywords:** anti-inflammatory activity, multimeric association, Bowman-Birk inhibitor, seed proteins, *Mucuna pruriens*

## Abstract

A Bowman-Birk protease, i.e., *Mucuna pruriens* trypsin inhibitor (MPTI), was purified from the seeds by 55.702-fold and revealed a single trypsin inhibitor on a zymogram with a specific activity of 202.31 TIU/mg of protein. On sodium dodecyl sulfate–polyacrylamide gel electrophoresis (SDS–PAGE) under non-reducing conditions, the protease trypsin inhibitor fraction [i.e., trypsin inhibitor non-reducing (TINR)] exhibited molecular weights of 74 and 37 kDa, and under reducing conditions [i.e., trypsin inhibitor reducing (TIR)], 37 and 18 kDa. TINR-37 revealed protease inhibitor activity on native PAGE and 37 and 18 kDa protein bands on SDS–PAGE. TINR-74 showed peaks corresponding to 18.695, 37.39, 56.085, and 74.78 kDa on ultra-performance liquid chromatography (UPLC) coupled with electrospray ionization/quadrupole time-of-flight-mass spectrometry (ESI/QTOF-MS). Similarly, TINR-37 displayed 18.695 and 37.39 kDa peaks. Furthermore, TIR-37 and TIR-18 exhibited peaks corresponding to 37.39 and 18.695 kDa. Multiple peaks observed by the UPLC-ESI/QTOF analysis revealed the multimeric association, confirming the characteristic and functional features of Bowman-Birk inhibitors (BBIs). The multimeric association helps to achieve more stability, thus enhancing their functional efficiency. MPTI was found to be a competitive inhibitor which again suggested that it belongs to the BBI family of inhibitors, displayed an inhibitor constant of 1.3 × 10^–6^ M, and further demonstrates potent anti-inflammatory activity. The study provided a comprehensive basis for the identification of multimeric associates and their therapeutic potential, which could elaborate the stability and functional efficiency of the MPTI in the native state from *M. pruriens*.

## Introduction

Protease inhibitors (PIs) are small proteins or peptides that inhibit the proteolytic activity of enzymes by forming high-affinity stoichiometric complexes (Bode and Huber, 2000). PIs are widely distributed in plants, animals, and microorganisms. Furthermore, they are mostly abundant in seeds and tubers of plants belonging to the Fabaceae, Solanaceae, Cucurbitaceae, and Poaceae families (Connors et al., 2002). PIs have been grouped into various classes, depending on their inhibitory specificity, of which serine-type PIs (SPIs) are most comprehensively studied and characterized (Rawlings et al., 2018). Moreover, several types of SPIs have been extensively purified, characterized, and assessed for their biological potential from various sources (Clemente et al., 2019). In plants, these proteins are involved in many physiological processes, such as the regulation of endogenous and exogenous proteolysis, delaying senescence, and acting as defensive molecules, thereby protecting against pathogens (Pak and Van Doorn, 2005; Joshi et al., 2014; Yasin et al., 2014, 2020). Additionally, they are involved in signal transduction, mobilization of storage proteins, and morphogenesis during plant development (Yasin et al., 2014, 2020; Rustgi et al., 2017).

Recently, SPIs have garnered substantial attention for potential application in biomedicine and biotechnology. They act as efficient tools to control unwanted proteolysis and to treat conditions, such as AIDS, respiratory diseases, hypertension, cancer, microbial infections, and neurodegenerative disorders, such as Alzheimer’s disease. Furthermore, SPIs regulate blood coagulation, inflammation, signal cascade in the immune system and cell cycle. Therefore, SPIs can be the potent candidates for drug design and development (Fumagalli et al., 1996; Thompson and Palmer, 1998; Kato, 1999; Kataoka et al., 2002; Nyberg et al., 2006; Srikanth and Chen, 2016; Cristina Oliveira de Lima et al., 2019; Kim et al., 2020). The Bowman-Birk inhibitor (BBI) family is a type of SPIs with the characteristic features of two reactive sites, independent and simultaneous inhibition of two serine proteases with different specificity, rigid compact structure, stability to extreme pH, and temperature conditions (Hellinger and Gruber, 2019). It is believed that dicot BBIs could have been evolved from a single-headed ancestral BBI *via* internal gene duplication, fusion, and mutation processes. This suggested that a higher molecular weight (MW) of 16 kDa BBIs might have been evolved from a small MW of 8 kDa BBIs (Song et al., 1999). This hypothesis gets credence by the presence of intramolecular sequence homology in BBIs. At present, the sequences of numerous BBIs from various plant sources are available at the plant PIs database accessible at the MEROPS database^1^ and the NCBI database.^2^ BBI has potential pharmaceutical properties and therapeutic applications (Hellinger and Gruber, 2019; Gitlin-Domagalska et al., 2020).

The association of SPIs in legumes is a natural, fundamental process. The appropriate association of the monomers to multimeric forms helps in the sustained/retained activity of the inhibitor in its native state. If the monomers do not associate and form the stable multimeric structure, the inhibitor is rendered inactive, thereby reducing its biological activity (Catalano et al., 2003). Reportedly, the BBI type commonly exhibits this kind of association (Arentoft et al., 1993; Prasad et al., 2010). Ultra-performance liquid chromatography-electrospray ionization/quadrupole time-of-flight-mass spectrometry (UPLC-ESI/QTOF-MS) is a high resolution, less elution time MS (in a column with a non-porous particle size below 2 mm) for proteins (Eschelbach and Jorgenson, 2006). Furthermore, UPLC is a pivotal tool for analyzing associated proteins in bulk coupled with good retention time, thereby more number of proteins with higher molecular mass can be efficiently observed (Everley and Croley, 2008). However, adequate information regarding the utilization of UPLC-based MS to identify the monomeric forms of BBIs is lacking. In this regard, we attempted to identify the association of monomeric forms of a BBI from *Mucuna pruriens* seeds through UPLC-ESI/QTOF-MS.

The genus *Mucuna* of the Fabaceae family includes numerous plants of diverse habitats. *M. pruriens* is an underutilized forage and medicinal plant primarily used by tribal communities of India, China, and African countries for treating snakebites as an antagonist to the venom (*Naja* spp., *Echis*, *Calloselasma*, and *Bungarus*). In addition, the plant has shown potential in prophylactic treatment (Guerranti et al., 1999; Tan et al., 2009; Meenatchisundaram and Michael, 2010). *M. pruriens* seeds can be used to cure Parkinson’s disease and depressive neurosis (Katzenschlager et al., 2004). Recent studies on *M. pruriens* resulted in successful isolation and purification of various biological compounds such as carboxylesterase and amylase inhibitor (Chandrashekharaiah et al., 2011; Bharadwaj et al., 2018). SPIs are putative targets in pharmaceutics; however, their multimeric associations are yet to be characterized. In view of their contemporary importance, the present investigation was undertaken to study SPIs from a potential source, i.e., *M. pruriens* seeds. This study illustrates the purification and characterization of a BBI and the multimeric associations of its monomers by UPLC-ESI/QTOF-MS.

## Materials and Methods

### Materials

The *M. pruriens* seeds were collected from the Indian Council of Agricultural Research, Indian Institute of Horticultural Research Center, Bangalore, India.

### Purification of *Mucuna pruriens* Trypsin Inhibitor

The *M. pruriens* seeds were soaked for 12 h at room temperature; the seed coats were removed; and a 10% butanol cake of the cotyledons was prepared (Wetter, 1957). The cake obtained was dried, powdered, and stored at 4°C until used. Furthermore, 10% crude “trypsin inhibitor” extract was prepared by stirring the powder in 0.1 N HCl on a magnetic stirrer at 4°C for 1 h, followed by centrifugation (Remi C-24 Plus, Vasai, India) at 10,000 rpm for 30 min at 4°C. Supernatant was collected and subjected to 0–25%, 25–50%, and 50–90% ammonium sulfate fractionation (Bollag et al., 1996). Pellets obtained after fractionation were dissolved separately in sodium acetate buffer (0.025 mM; pH 5.7) and dialyzed for 1.3 h against the same buffer at 4°C. A fraction containing the 25–50% dialysate was loaded onto the carboxymethyl cellulose (CM)-cellulose column (2.3 × 7.5 cm) preequilibrated with sodium acetate buffer (0.025 mM; pH 5.7). The column was washed with start buffer (0.025 mM sodium acetate buffer; pH 5.7) at a flow rate of 60 ml/h with a fraction volume of 10 ml. Bound proteins were eluted stepwise using 0.1, 0.3, and 0.5 M NaCl. The column-bound fraction exhibiting trypsin inhibitor activity was pooled and concentrated. Furthermore, the concentrated CM-cellulose fraction was subjected to Sephadex G-75 gel filtration chromatography (1.0 × 1.5 cm) preequilibrated with sodium acetate buffer (0.025 mM; pH 5.7). Fractions of 2 ml were collected at a flow rate of 10 ml/h. Fractions exhibiting trypsin inhibitor activity were pooled and concentrated using Centricon tubes (MilliporeMerck, Darmstadt, Germany) (MW cutoff of 5 kDa). The concentrated Sephadex G-75 fraction was further subjected to preparative gel electrophoresis as follows: 10% non-denaturing preparative cationic PAGE performed at pH 4.3 (10% T and 5% C) (Reisfeld et al., 1962). Following electrophoresis, a native zymogram was performed to identify bands containing trypsin inhibitor activity; such bands were sliced, transferred, and macerated in a glass homogenizer at 4°C, followed by centrifugation at 5,000 rpm for 10 min. The supernatant obtained was stored for further analysis.

### Protein Estimation

Total protein was determined as reported previously, using bovine serum albumin (BSA) as standard (Lowry et al., 1951). The protein content of all the CM-cellulose fractions was quantified by measuring the absorbance at 280 nm using a UV-Visible spectrophotometer serine protease (SP 3000-Plus, Optima, Tokyo, Japan).

### Trypsin Activity and Trypsin Inhibitory Activity

Trypsin activity assay was performed using N-Benzoyl-DL-arginine-4-nitroanilide hydrochloride (BAPNA) as the substrate (Shibata et al., 1986). Trypsin was dissolved in 0.001 N HCl containing 20 mM CaCl_2_ at a concentration of 200 μg/ml. BAPNA (200 mg) was dissolved in 3 ml dimethyl sulfoxide (DMSO), and the volume was made up to 100 ml with 0.1 M Tris–HCl buffer (pH 8.2). The assay mixture (containing 500 μl trypsin, 500 μl 0.1 M Tris–HCl buffer pH 8.2, and 1.25 ml BAPNA) was incubated at 37°C for 15 min. The reaction was terminated by the addition of 0.4 ml of 30% acetic acid. The absorbance was read at 410 nm against reagent blank and converted to trypsin unit (TU; one TU increases optical density (OD) by 0.01 at 410 nm).

Trypsin inhibitory activity was measured as reported previously (Shibata et al., 1986). The assay mixture (500 μl trypsin, 400 μl 0.1 M Tris–HCl buffer pH 8.2, and 100 μl purified inhibitor) was incubated at 37°C for 15 min followed by the addition of 1.25 ml BAPNA and again incubated for 10 min at 37°C. The reaction was terminated by the addition of 0.4 ml of 30% acetic acid. The absorbance was measured at 410 nm against reagent blank and converted to trypsin inhibitor unit (TIU; one TIU decreases OD by 0.01 at 410 nm).

### Electrophoresis

Cationic native PAGE was performed with 10% T and 5% C as previously described (Reisfeld et al., 1962). After electrophoresis, the gel was stained for proteins with 0.1% Coomassie brilliant blue G-250 (w/v) in 3.5% (w/v) perchloric acid for 1 h and washed several times with distilled water. The gel was destained with 25% methanol and 10% acetic acid solution.

The inhibitory activity was quantified by reverse zymography on gelatin-PAGE, wherein 1% gelatin was copolymerized with the polyacrylamide matrix (Felicioli et al., 1997). After electrophoresis, the gel was incubated with Tris–HCl buffer (0.1 M; pH 7.6) for 30 min, followed by trypsin treatment (100 μg/ml) for 30 min. The gel was washed with distilled water, stained, and destained as prescribed earlier (Felicioli et al., 1997). Colored bands appeared against a transparent background correspond to trypsin inhibition.

The SDS–PAGE was carried out using a 12.5% polyacrylamide gel (12.5% T and 5% C) with or without β-mercaptoethanol (β-ME) (Laemmli, 1970). After the electrophoretic run, the gel was removed, stained, and destained as described earlier. The non-reducing band corresponding to 37 kDa (TINR-37) was sliced, transferred, and macerated in a glass homogenizer at 4°C followed by centrifugation at 5,000 rpm for 10 min. The supernatant obtained was subjected to native PAGE for both protein and inhibitor staining. The homogeneity of the purified trypsin inhibitor (TINR-37) was analyzed on the SDS–PAGE in the presence or absence of β-ME, as described earlier.

### Ultra-Performance Liquid Chromatography-Electrospray Ionization/Quadrupole Time-of-Flight-Mass Spectrometry Analysis

The SDS was removed from both non-reducing and reducing SDS–PAGE gels by treating them with Triton X-100. PI bands [i.e., *M. pruriens* trypsin inhibitor (MPTI)] corresponding to the MWs of 74 and 37 kDa from the non-reducing gel (i.e., TINR-74 and TINR-37) and that of 37 and 18 kDa from the reducing gel (i.e., TIR-37 and TIR-18) were sliced and transferred to a glass homogenizer separately. The gels were homogenized at 4°C with sodium acetate buffer (0.025 M; pH 5.7) with an equal volume of 1 M CaCl_2_ (Zhou et al., 2012). The homogenate was centrifuged at 10,000 × *g* for 10 min, and the supernatant was collected and filtered using Whatman No. 1 filter papers. The filtrates were analyzed using UPLC (Acquity UPLC, Waters Corporation, Milford, CT, United States) coupled with ESI/QTOF-MS (Synapt G2-Si, Waters Corporation, Milford, CT, United States). Separations were carried out using an ethylene-bridged hybrid C4 column (2.1 × 50 mm, 1.7 μm) with 0.1% formic acid in double distilled water and acetonitrile as the mobile phase. All acquisitions were performed on positive polarity with the following ESI settings: capillary voltage = 3 kV, cone voltage = 40 V, source temperature = 120°C, desolvation gas = 350°C, and the instrument was tuned for protein analysis. The MS data were acquired with a mass range from 400 to 4,000 m/z on the UPLC-based ESI/QTOF-MS. The instrument was operating on a resolution of 40,000 full width at half maximum (FWHM). The charge state spectra were subject to deconvolution using the maximum entropy 1 algorithm on the UVIFI informatics platform (Wang et al., 2018).

### Determination of k_*m*_, V_*max*_, and Inhibitor Constant (k_*i*_)

The k_*m*_ and V_*max*_ of trypsin for BAPNA in the presence of MPTI were determined from the Lineweaver–Burk plot; the inhibition constant (k_*i*_) was calculated from the plots (Lineweaver and Burk, 1934). The residual inhibitory activities were determined using BAPNA as a substrate, as described earlier.

### Determination of Anti-inflammatory Activity Using the Hemolytic Assay

The hemolytic assay with slight modifications was performed (Mizushima and Kobayashi, 1968). Goat blood samples (20 ml at 0–4°C) were collected using anticoagulants from a slaughterhouse. The suspension solution of red blood cells (RBC) was prepared by centrifuging the blood sample at 3,000 rpm for 5 min at 4°C, followed by three washes with 0.85% NaCl solution. The volume of the suspension was made up to 20 ml with isosaline. The assay mixture of 1 ml (100, 200, 300, and 400 μg) of MPTI with 2 ml hyposaline and 0.5 ml RBC suspension was incubated for 30 min and then centrifuged at 1,500 rpm for 10 min. The supernatant was collected, and its absorbance was measured at 560 nm using a spectrophotometer. Control was prepared using hyposaline and the RBC suspension. Diclofenac (1 mg/ml) was used as the standard drug.


(1) %inhibitionofhemolysis=100×(1-A2/A1),

where A1 = absorption of control and A2 = absorption of test sample.

### Statistical Analysis

All experiments were performed in replicates. The data were analyzed using SAS 9.2 (SAS Institute Inc., Cary, NC, United States) and Excel. The data shown are mean ± SE/SD. Results are expressed as graphs prepared using GraphPad Prism 5 software (GraphPad Software, Inc., California, United States) and Microsoft Excel.

## Results

### Purification Analysis

**FIGURE 1:**
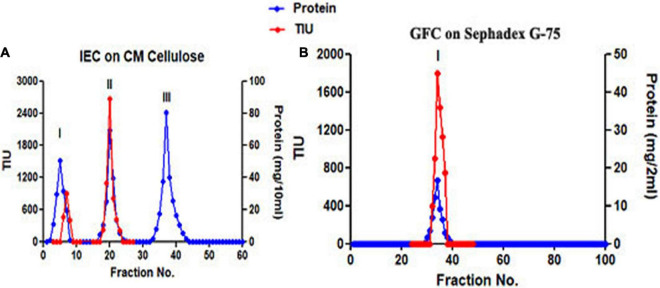
Separation of protease inhibitors (PIs) during ion exchange and gel filtration chromatography. **(A)** Elution profile on CM-cellulose chromatography and **(B)** elution profile on Sephadex G-75 chromatography.

**FIGURE 2:**
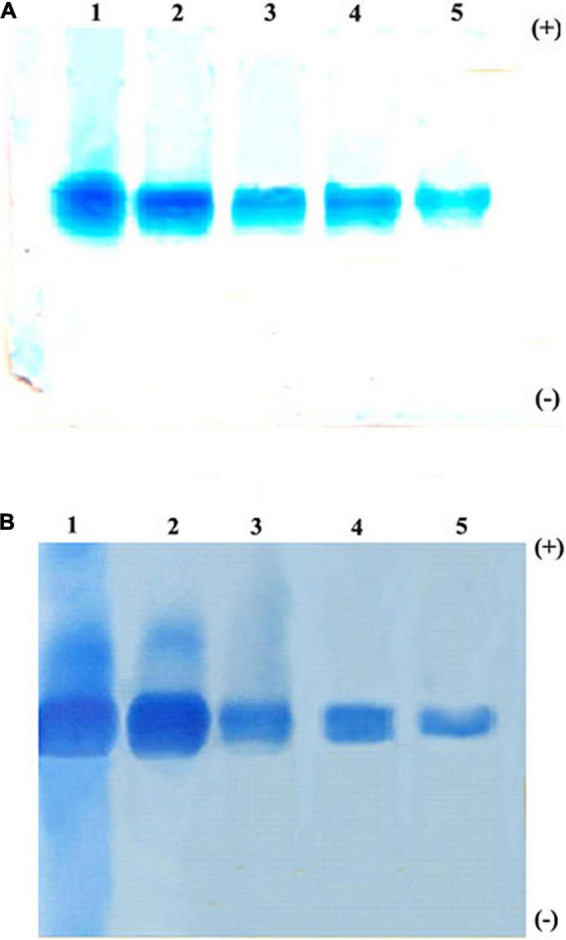
Native polyacrylamide gel electrophoresis (PAGE) pattern of proteins and PI profile of *Mucuna pruriens* seeds. **(A)** Protein staining and **(B)** protease inhibitor(PI) staining. Lanes in gel: 1. crude inhibitor extract, 2. ammonium sulfate fraction (25–50%), 3. CM-cellulose fraction-II, 4. Sephadex G-75 fraction, and 5. preparative PAGE fraction.

The trypsin-specific PI was purified to homogeneity from *M. pruriens* seeds using different conventional techniques such as acid extraction, salt fractionation, CM-cellulose chromatography, Sephadex G-75 gel filtration chromatography, and preparative gel electrophoresis. The purification results showing recovery, fold purification, and specific activity at each stage are given in Table 1. The crude PI extract was subjected to 0–25% and 25–50% ammonium sulfate fractionation. The pellet obtained from 25 to 50% saturation was dialyzed and subjected to CM-cellulose chromatography. The elution profile of CM-cellulose chromatography is shown in Figure 1A. Three protein peaks were eluted and designated as Fraction-I, -II, and -III. Fraction-I was not adsorbed onto the CM-cellulose column at pH 5.7 and hence eluted along the start buffer. Fraction-II and Fraction-III were eluted with 0.1 and 0.3 M NaCl in the start buffer at pH 5.7. Fraction-II containing appreciable levels of PI activity was pooled, concentrated, and subjected to Sephadex G-75 gel filtration chromatography. The elution profile of Sephadex G-75 chromatography at pH 5.7 is shown in Figure 1B. Both the protein and PI were eluted together in a single fraction, followed by subjecting this fraction to preparative PAGE. Fold purification of the resultant PI on preparative PAGE was nearly 56%, whereas its specific inhibitor activity was 202.31 TIU/mg (Table 1). The analysis of the purification profile of the PI on reverse zymography and cationic native PAGE showed a single PI activity band corresponding to a single protein band (Figures 2A,B).

**TABLE 1:** Purification profile of cationic serine protease inhibitor from *Mucuna pruriens* seeds.

Purification steps	Protein (mg)	TIU	Specific inhibitory activity (TIU/mg)	% yield	Fold of purification
Crude	4735.5	17,200	3.6	100	1
ASF 25–50%	571.3	7,200	10.7	42	3
CM-cellulose chromatography	170.5	5,430	32	31.6	8.7
Sephadex G-75 chromatography	50.1	3,680	73.5	21.4	20.2
Preparative PAGE (MPTI)	5.2	1,050	202.3	6.1	55.7

*TIU, Trypsin inhibitor units; ASF, ammonium sulfate fraction; MPTI, Mucuna pruriens trypsin inhibitor; PAGE, polyacrylamide gel electrophoresis.*

### Electrophoretic Analysis: Criteria of Purity

**FIGURE 3:**
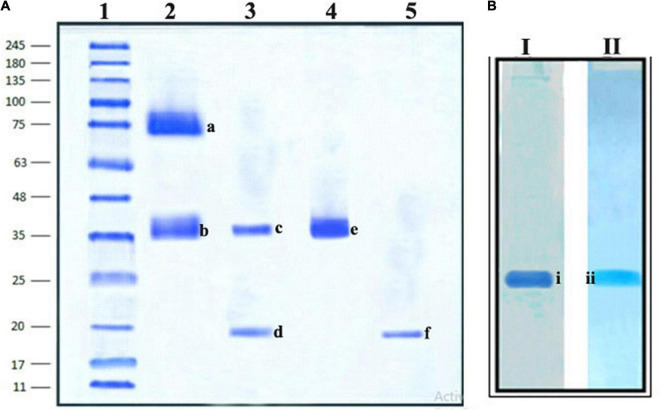
Sodium dodecyl sulfate (SDS)–PAGE and Native PAGE of *Mucuna pruriens* trypsin inhibitor (MPTI). **(A)** SDS–PAGE of preparative PAGE fraction. Lanes: 1. standard marker proteins (kDa), 2. preparative PAGE fraction (non-reducing), 3. preparative PAGE fraction (reducing), 4. SDS–PAGE fraction b (non-reducing), and 5. SDS–PAGE fraction b (reducing). a: Trypsin inhibitor non-reducing (TINR)-74 kDa, b and e: TINR-37 kDa, c: trypsin inhibitor reducing (TIR)-37 kDa, d and f: TIR-18 kDa. **(B)** Native PAGE of SDS–PAGE fraction, (i) native protein PAGE and (ii) native zymogram PAGE.

The homogeneity of the purified trypsin inhibitor was established using native PAGE and SDS–PAGE. The preparative PAGE fraction was subjected to SDS–PAGE under both non-reducing and reducing conditions. The non-reducing SDS–PAGE showed the presence of two protein bands corresponding to 74 kDa (TINR-74) and 37 kDa (TINR-37) bands. In contrast, the reducing SDS–PAGE showed the presence of two protein bands corresponding to 37 (TIR-37) and 18 kDa (TIR-18). TINR-37 band was eluted from the gel and further subjected to SDS–PAGE under both reducing and non-reducing conditions. Accordingly, the presence of 37 and 18 kDa proteins in the non-reducing and reducing gels, respectively, confirmed the dimeric nature of the purified PI. Cationic native PAGE of the TINR-37 inhibitor showed a single PI activity corresponding to a single protein band (Figures 3A,B).

### Determination of k_*m*_, V_*max*_, and k_*i*_

**FIGURE 4:**
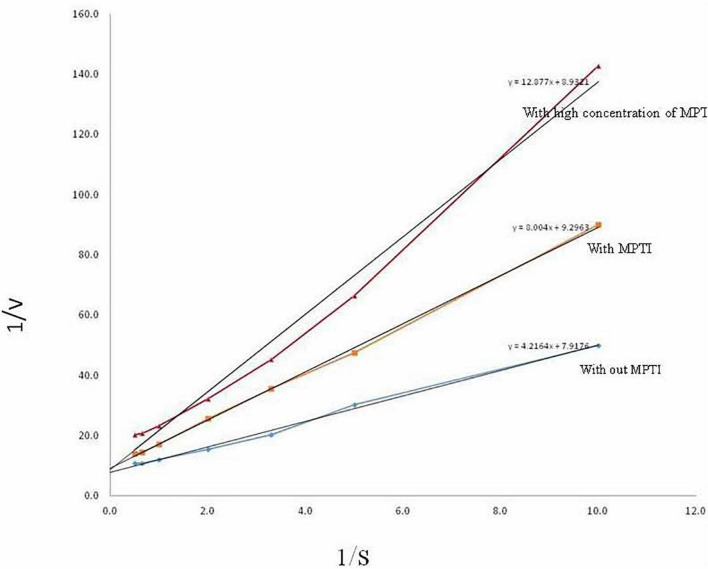
Determination of k_*m*_ and V_*max*_ of trypsin in the presence of *Mucuna pruriens* trypsin inhibitor (MPTI).

The inhibitory activity of MPTI was determined against bovine trypsin by measuring the residual activity toward BAPNA as a substrate. k_*m*_ and V_*max*_ of trypsin were determined as 0.126 mM and 0.532 μmol/min, respectively, using the Lineweaver–Burk plots (Figure 4). The altered k_*m*_ and V_*max*_ of trypsin at low and high concentrations of MPTI were 0.862 and 1.443 mM, as well as 0.108 and 0.113 μmol/min, respectively. The increased k_*m*_ in the presence of MPTI indicated that MPTI acts as a competitive inhibitor. k_*i*_ was found to be 1.3 × 10^–6^ M.

### Characterization of Multimeric Forms of Purified TI Based on Ultra-Performance Liquid Chromatography-Electrospray Ionization/Quadrupole Time-of-Flight-Mass Spectrometry

**FIGURE 5:**
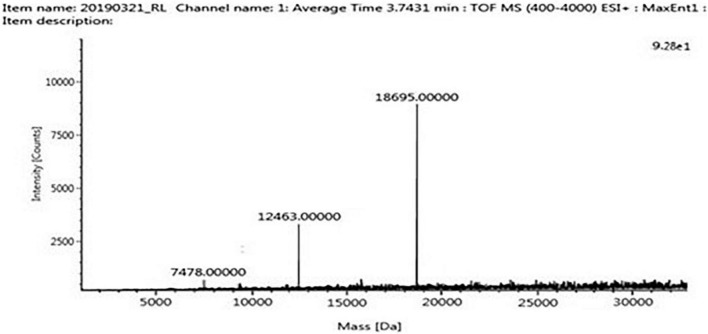
Ultra-performance liquid chromatography-electrospray ionization/quadrupole time-of-flight-mass spectrometry (UPLC-ESI/QTOF-MS) analysis of TIR-18.

**FIGURE 6:**
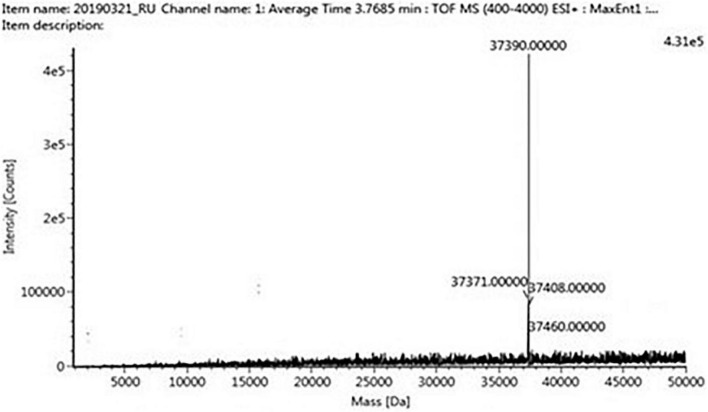
Ultra-performance liquid chromatography-electrospray ionization/quadrupole time-of-flight-mass spectrometry (UPLC-ESI/QTOF-MS) analysis of TIR-37.

**FIGURE 7:**
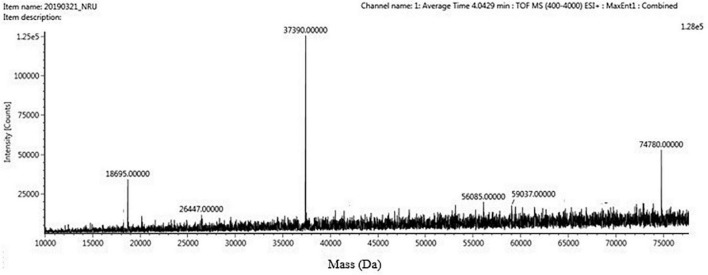
Ultra-performance liquid chromatography-electrospray ionization/quadrupole time-of-flight-mass spectrometry (UPLC-ESI/QTOF-MS) analysis of TINR-74.

**FIGURE 8:**
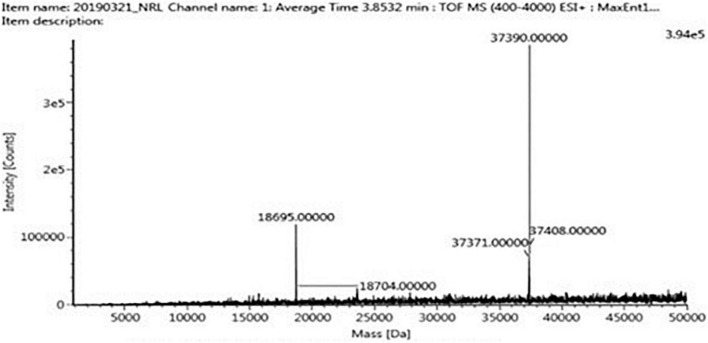
Ultra-performance liquid chromatography-electrospray ionization/quadrupole time-of-flight-mass spectrometry (UPLC-ESI/QTOF-MS) analysis of TINR-37.

The four trypsin inhibitor bands (i.e., TINR-74, TINR-37, TIR-37, and TIR-18) were extracted from the electrophoretic gels and subjected to UPLC-ESI/QTOF-MS. TIR-18 and TIR-37 revealed the presence of 37.39 and 18.695 kDa peaks (Figures 5, 6), respectively. TINR-74 was observed to contain multiple peaks of varying MWs, i.e., 18.695, 37.39, 56.085, and 74.78 kDa (Figure 7), whereas TINR-37 revealed the presence of peaks at 18.695 and 37.39 kDa (Figure 8). These results indicated that the purified PI fraction showed an association of multimeric forms under native conditions.

### Anti-inflammatory Activity

The purified MPTI was used to test the stabilization of the RBC membrane, whereby MPTI showed maximum stabilization at a concentration of 300 μg/ml MPTI (Supplementary Figure 1). This revealed that the purified PI can act as a potent anti-inflammatory agent, which indicates that the presence of multimeric association may assist in increased efficiency and stability to the MPTI.

## Discussion

The PIs are the proteolytic enzyme inhibitors reported from several legumes and explored for many applications in agriculture and biotechnology (Srikanth and Chen, 2016; Clemente et al., 2019). *M. pruriens*, being an underutilized legume, is rich in protein and contains various bioactive molecules such as PIs (Lone et al., 2021). Although PI is present, the acid-resistant PI with its multimeric forms is yet to be explored. On this basis, the proteinaceous PI was purified and characterized from *M. pruriens*.

The PI was isolated from *M. pruriens* by acid extraction and partially purified by ammonium sulfate precipitation. It was found that 30–60% ammonium sulfate saturation obtained here contained the highest content of protease inhibitory activity. Previously, PIs (30–40 kDa) have been fractionated with 50% ammonium sulfate saturation from the Australian wattle seed *Acacia victoriae* Bentham (Ee et al., 2008). Further purification was performed by employing CM-cellulose and Sephadex G-75 chromatography. PI with an MW of 43 kDa was isolated and purified from duck egg albumin using salt fractionation, affinity, and gel filtration chromatography techniques (Quan and Benjakul, 2019). Protein recovery and fold can be increased by combining different purification methods. The fold purification obtained in this study is comparatively higher when compared to *Acacia nilotica* seeds (Babu et al., 2012). PIs reported from various legumes ranged from 19- to 489-fold purification level (Godbole et al., 1994; Hajela et al., 1999; Oliveira et al., 2002; Rai et al., 2008).

The purification of PI from the seeds of *M. pruriens* showed satisfactory specific activity when isolated through acid extraction, and during all stages of the purification, it revealed a single protein band on native PAGE with their corresponding trypsin inhibitor on native reverse zymography PAGE, which confirmed that only acid-resistant PI is present. SDS–PAGE under non-reductive and reducing conditions revealed the MWs of 37 and 18 kDa of purified PI, which confirmed the presence of monomer and dimeric forms. Earlier, a 34-kDa PI has been characterized using SDS–PAGE from *Putranjiva roxburghii* seeds previously (Chaudhary et al., 2008). The presence of this self-association to form multimers was identified by UPLC-ESI/QTOF-based MS. This analysis confirmed that the purified MPTI exhibited multimeric forms that are essential for its molecular packing and assembly as a storage protein in seeds (Chaudhary et al., 2008; Prasad et al., 2010). In addition, these analyses showed that the purified MPTI fraction belongs to the BBI family. BBIs are well-known to undergo spontaneous self-associations to form homodimers, trimers, or even more complex oligomers, which constitute multimeric forms (Gennis and Cantor, 1976; Odani and Ikenaka, 1978; Terada et al., 1994; Catalano et al., 2003; Kumar et al., 2004, 2015; Silva et al., 2005; Rao and Suresh, 2007; Brand et al., 2017). Traditionally, the BBIs have rich cysteine residues and have multiple hydrophobic groups, which favor them in forming various intermolecular interactions. Such interactions exist in their native state. The light-scattering method has been used to establish the association of monomers and several multimers (i.e., dimers, trimers, tetramers, and hexamers) in BBIs (de Freitas et al., 1997). The association of subunits in BBIs is due to a strong network of hydrogen bonds between the buried charged residues and exposed hydrophobic surface patches; this helps in the self-association of the protein and stabilizes the disulfide bonds by forming an electrically charged, constrained, rigid monomeric structure (Silva et al., 2005; Rao and Suresh, 2007; Joshi et al., 2013). Some studies reported the pivotal role of charged interactions in the case of monomer/dimer equilibria (Kumar et al., 2004). During the MS analyses, the non-covalent interactions break down, resulting in separate peaks, thereby confirming the multimeric association. It has been reported that the multimeric association between peptides/proteins is due to the formation of ionic bonding between Ca^2+^ ions and glutamate (E) and aspartate (D) residues or hydrogen bonding between hydroxyl groups (OH) of threonine (T) and/or serine (S) with the neighboring or asparagine (N) and tyrosine (Y) residues. Moreover, peptides/proteins with more number of asparagine (N) and tyrosine (Y) residues can help in increasing binding potential (Jamalian et al., 2014). In horse gram, the stabilization of dimers is due to the electrostatic interaction between Lys-24 and Asp-75 = 76 residues of N- and C-terminal ends (Kumar et al., 2004; Muricken and Gowda, 2010). However, the mechanism of self-association may vary among various BBI molecules. The multimeric states of trypsin inhibitor were too identified by the atomic force microscopy analysis, which indicated that the inhibitor adopts stable and well-packed self-associated states in monomer-dimer-trimer-hexamer forms with globular-ellipsoidal shapes (Silva et al., 2005). The absence of a more hydrophobic core and the presence of the high content of disulfide bonds result in a constrained conformation that may be responsible for the remarkable stability and association exhibited by this inhibitor (da Silva et al., 2001), in covenant with other BBIs (Voss et al., 1996). It has been reported that the reactive sites that show interaction with proteases are generally located on the surface of the dimer, which most likely indicates that the dimeric form is the functional state of the molecule (Li de la Sierra et al., 1999). The multimeric association shown by the BBI type of inhibitors is a natural fundamental characteristic feature that helps them to achieve more stability, thus enhancing their functional efficiency. Due to this association, BBIs are more resistant to extreme acidic extraction. Furthermore, the self-association tendency to form the multimeric forms is mostly related to the physiological function of BBI as it acts as a plant storage protein, which facilitates their tight packing in seeds (Catalano et al., 2003; Hogg, 2003; Kumar et al., 2015). In this study, UPLC coupled with ESI/QTOF-MS confirmed the multimeric association of purified SPI belonging to the BBI family of PIs.

The molecular masses determined by both SDS–PAGE and UPLC-ESI/QTOF-MS were found higher as compared to reported BBIs. This is in accordance with those found for BBIs from other species (Gladysheva et al., 1994; Yan et al., 2009; Chevreuil et al., 2014; Al-Maiman et al., 2019). Regarding the existence of multiple forms or higher MWs, it may be possible that this happens due to the evolution through gene duplication, where it has been reported that 16 kDa inhibitor was evolved by gene duplication from 8 kDa inhibitor (Song et al., 1999). This suggested that the purified PI from the seeds of *M. pruriens* is a novel BBI.

Furthermore, the kinetic analysis suggests that MPTI forms a more stable complex with trypsin by increased k_*m*_ in the presence of MPTI, which indicated that MPTI acts as a competitive inhibitor. The tendency of forming a high-affinity complex toward trypsin interestingly indicated that most BBI belongs to competitive inhibition. MPTI exhibited a k_*i*_ value of 1.3 × 10^–6^ M. PIs with k_*i*_ values ranging between 0.0001 and 5.2 mM are the characteristics of BBIs (Prasad et al., 2010). Earlier, it was reported that the k_*i*_ value of PIs from different plant sources (5.3 × 10^–10^ M in *Dimorphandra mollis*, 1.7 × 10^–9^ M in *D. mollis*, 2.5 × 10^–10^ M in *Archidendron ellipticum*, and 1.4 × 10^–11^ M in *P. roxburghii* seeds) was quite less as compared to MPTI (Macedo et al., 2000; Mello et al., 2001; Bhattacharyya et al., 2006; Chaudhary et al., 2008).

Proteases, being clinically important molecules, are putative drug targets among which serine proteases comprise a major portion. Model SPs such as chymotrypsin, trypsin, pancreatic elastase, or several blood coagulation factors became the hotspots for the drug target and discovery of PIs (López-Otín and Matrisian, 2007; Drag and Salvesen, 2010). Most of the animal proteases are still unexplored as drug targets. Several studies have reported that various proteases are involved in arthritic reactions and tissue damage during inflammation. Among them, serine protease is the most actively involved in inflammation, causing the stimulation of eosinophils through protease-activated receptor-2 (PAR2) response (King et al., 2000; Miike et al., 2001). On this basis, we analyzed the role of MPTI in inhibiting the membrane disruption on RBC hemolysis. It was found that MPTI exhibited potent anti-inflammatory activity. BBIs have been used to cure the inflammatory disorders of the gastrointestinal tract, which manifest that the chronic inflammation of tissues results in elevating the activity of plasma proteases (Juritsch and Moreau, 2018). In addition, inflammation can also be caused by the denaturation of proteins. The anti-inflammatory drugs, such as phenylbutazone and salicylic acid, have been reported, which inhibit protein denaturation (Selvi and Bhaskar, 2012). It has also been studied that Plant PIs may inhibit the action of proteinases, bactericidal enzymes, and neutrophils released from lysosomes at the site of inflammation, which, on extracellular release, cause further tissue inflammation and damage (Armstrong, 2001; Pham, 2006). Therefore, this extracellular inhibition of the protease can help in directing specific drug targets and will result in the development of novel natural drug therapeutics.

## Conclusion

The UPLC-ESI/QTOF-MS is a powerful technique for the exact mass calculation of proteins. The cationic SPI was identified, purified, and characterized from the seeds of an underutilized legume, i.e., *M. pruriens* employing acid extraction, salt precipitation, CM-cellulose, Sephadex G-75 chromatography, preparative native, and SDS–PAGE. The homogeneity of the purified inhibitor was established by native and SDS–PAGE. The MW of the purified inhibitor was determined to be 18.3 kDa in monomeric form and 37.63 kDa in dimeric form, *via* UPLC-ESI/QTOF-MS. Furthermore, the multimeric association of the purified PI was characterized by UPLC-ESI/QTOF-MS. This self-association may arise due to non-covalent interactions between the intact proteins. This characterization study revealed the association of multimeric forms of cationic trypsin inhibitors in the seeds of *M. pruriens*, which specified its nature toward the BBI family. Furthermore, the purified inhibitor was determined as a competitive inhibitor and exhibited potent anti-inflammatory activity. Apart from pest resistance in plants, purified MPTI can be used in the medical field to evaluate its potential as a curative measure for inflammatory diseases, cardiovascular diseases, and cancer.

## Data Availability Statement

The original contributions presented in the study are included in the article/Supplementary Material, further inquiries can be directed to the corresponding authors.

## Author Contributions

JL conducted the research and wrote the manuscript. ML, RB, and FA performed the data analyses and reviewed the manuscript. SW, MP, and JY edited and reviewed the manuscript. KC planned, supervised, and organized the experiment and reviewed the manuscript. All authors contributed to the article and approved the submitted version.

## Conflict of Interest

The authors declare that the research was conducted in the absence of any commercial or financial relationships that could be construed as a potential conflict of interest.

## Publisher’s Note

All claims expressed in this article are solely those of the authors and do not necessarily represent those of their affiliated organizations, or those of the publisher, the editors and the reviewers. Any product that may be evaluated in this article, or claim that may be made by its manufacturer, is not guaranteed or endorsed by the publisher.

## Footnotes

1
https://www.ebi.ac.uk/merops

2
https://www.ncbi.nlm.nih.gov

## Supplementary Material

Supplementary Figure 1 Anti-inflammatory activity of MPTI.

## Abbreviations

PI: Protease inhibitor
SPIs: serine protease inhibitors
BBI: Bowman-Birk inhibitor
MPTI: *Mucuna pruriens* trypsin inhibitor
TIR: trypsin inhibitor reducing
TINR: trypsin inhibitor non-reducing
SDS: sodium dodecyl sulfate
PAGE: polyacrylamide gel electrophoresis
UPLC-ESI/QTOF-MS: ultra-performance liquid chromatography-electrospray ionization/quadrupole time-of-flight-mass spectrometry
CaCl_2_: calcium chloride
HCl: hydrochloric acid
k_*m*_: substrate concentration
V_*max*_: velocity maximum
RBC: red blood cells.
