# Supplementary figures and images for: Multimeric Association of Purified Novel Bowman-Birk Inhibitor From the Medicinal Forage Legume Mucuna pruriens (L.) DC
Source: Front Plant Sci. 2021 Nov 25;12:772046. doi: 10.3389/fpls.2021.772046 (PMC8655843; doi:10.3389/fpls.2021.772046)

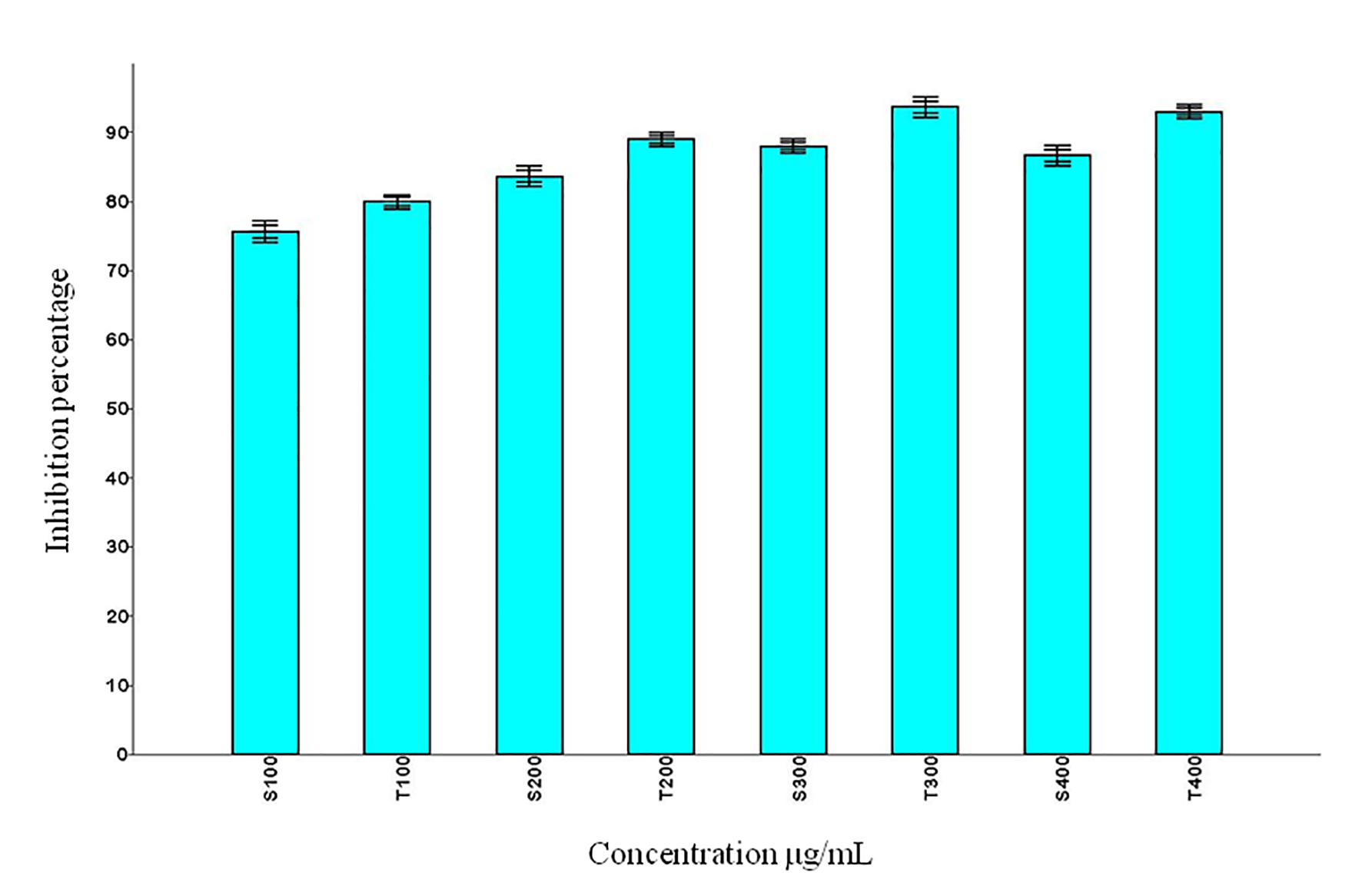

Supplement: Supplementary Figure 1 — Anti-inflammatory activity of MPTI. [file Image_1.TIF]
